# Correction: Hassel et al. Medical Needs and Therapeutic Options for Melanoma Patients Resistant to Anti-PD-1-Directed Immune Checkpoint Inhibition. *Cancers* 2023, *15*, 3448

**DOI:** 10.3390/cancers17233726

**Published:** 2025-11-21

**Authors:** Jessica C. Hassel, Lisa Zimmer, Thomas Sickmann, Thomas K. Eigentler, Friedegund Meier, Peter Mohr, Tobias Pukrop, Alexander Roesch, Dirk Vordermark, Christina Wendl, Ralf Gutzmer

**Affiliations:** 1 Skin Cancer Center, Department of Dermatology and National Center for Tumor Diseases (NCT), University Hospital Heidelberg, 69120 Heidelberg, Germany; jessica.hassel@med.uni-heidelberg.de; 2 Department of Dermatology, University Hospital Essen, 45147 Essen, Germany; lisa.zimmer@uk-essen.de (L.Z.); alexander.roesch@uk-essen.de (A.R.); 3 German Cancer Consortium (DKTK), Partner Site Essen, 69120 Heidelberg, Germany; 4 Bristol-Myers Squibb GmbH & Co. KGaA, 80636 Munich, Germany; thomas.sickmann@bms.com; 5 Department of Dermatology, Venereology and Allergology, Charité—Universitätsmedizin Berlin, Corporate Member of Freie Universität Berlin and Humboldt-Universität zu Berlin, 10117 Berlin, Germany; thomas.eigentler@charite.de; 6 Department of Dermatology, Skin Cancer Center at the University Cancer Centre and National Center for Tumor Diseases, Faculty of Medicine and University Hospital Carl Gustav Carus, Technical University Dresden, 01062 Dresden, Germany; friedegund.meier@uniklinikum-dresden.de; 7 Department of Dermatology, Elbe-Kliniken, 21614 Buxtehude, Germany; peter.mohr@elbekliniken.de; 8 Department of Internal Medicine III, Hematology and Oncology, University Hospital Regensburg, 93053 Regensburg, Germany; tobias.pukrop@klinik.uni-regensburg.de; 9 Bavarian Cancer Research Center (BZKF), 93053 Regensburg, Germany; 10 Department for Radiation Oncology, Martin-Luther University Halle-Wittenberg, 06108 Halle, Germany; dirk.vordermark@uk-halle.de; 11 Department of Radiology, University Hospital Regensburg, 93053 Regensburg, Germany; christina.wendl@ukr.de; 12 Department of Dermatology, Johannes Wesling Medical Center, Ruhr University Bochum, 32429 Minden, Germany

## Additional Affiliations

There was an error regarding the affiliations for Alexander Roesch. In addition to affiliation 2, the updated affiliations number should include: 2,3.

## Reference List

In the original publication [1], there was a mistake with the reference list. The correct reference list appears below. The scientific conclusions are unaffected. This correction was approved by the Academic Editor. The original publication has also been updated.

1. Sung, H.; Ferlay, J.; Siegel, R.L.; Laversanne, M.; Soerjomataram, I.; Jemal, A.; Bray, F. Global Cancer Statistics 2020: GLOBOCAN Estimates of Incidence and Mortality Worldwide for 36 Cancers in 185 Countries. *CA Cancer J. Clin.* **2021**, *71*, 209–249. https://doi.org/10.3322/caac.21660.
2. Schadendorf, D.; van Akkooi, A.C.J.; Berking, C.; Griewank, K.G.; Gutzmer, R.; Hauschild, A.; Stang, A.; Roesch, A.; Ugurel, S. Melanoma. *Lancet* **2018**, *392*, 971–984. https://doi.org/10.1016/S0140-6736(18)31559-9.
3. Michielin, O.; van Akkooi, A.C.J.; Ascierto, P.A.; Dummer, R.; Keilholz, U.; The ESMO Guidelines Committee. Cutaneous melanoma: ESMO Clinical Practice Guidelines for diagnosis, treatment and follow-updagger. *Ann. Oncol.* **2019**, *30*, 1884–1901. https://doi.org/10.1093/annonc/mdz411.
4. Garbe, C.; Amaral, T.; Peris, K.; Hauschild, A.; Arenberger, P.; Bastholt, L.; Bataille, V.; Del Marmol, V.; Dreno, B.; Fargnoli, M.C.; et al. European consensus-based interdisciplinary guideline for melanoma. Part 2: Treatment—Update 2019. *Eur. J. Cancer* **2020**, *126*, 159–177. https://doi.org/10.1016/j.ejca.2019.11.015.
5. Topalian, S.L.; Hodi, F.S.; Brahmer, J.R.; Gettinger, S.N.; Smith, D.C.; McDermott, D.F.; Powderly, J.D.; Sosman, J.A.; Atkins, M.B.; Leming, P.D.; et al. Five-Year Survival and Correlates Among Patients with Advanced Melanoma, Renal Cell Carcinoma, or Non-Small Cell Lung Cancer Treated with Nivolumab. *JAMA Oncol.* **2019**, *5*, 1411–1420. https://doi.org/10.1001/jamaoncol.2019.2187.
6. Robert, C.; Ribas, A.; Schachter, J.; Arance, A.; Grob, J.J.; Mortier, L.; Daud, A.; Carlino, M.S.; McNeil, C.M.; Lotem, M.; et al. Pembrolizumab versus ipilimumab in advanced melanoma (KEYNOTE-006): Post-hoc 5-year results from an open-label, multicentre, randomised, controlled, phase 3 study. *Lancet Oncol.* **2019**, *20*, 1239–1251. https://doi.org/10.1016/S1470-2045(19)30388-2.
7. Larkin, J.; Chiarion-Sileni, V.; Gonzalez, R.; Grob, J.J.; Rutkowski, P.; Lao, C.D.; Cowey, C.L.; Schadendorf, D.; Wagstaff, J.; Dummer, R.; et al. Five-Year Survival with Combined Nivolumab and Ipilimumab in Advanced Melanoma. *N. Engl. J. Med.* **2019**, *381*, 1535–1546. https://doi.org/10.1056/NEJMoa1910836.
8. Ugurel, S.; Rohmel, J.; Ascierto, P.A.; Becker, J.C.; Flaherty, K.T.; Grob, J.J.; Hauschild, A.; Larkin, J.; Livingstone, E.; Long, G.V.; et al. Survival of patients with advanced metastatic melanoma: The impact of MAP kinase pathway inhibition and immune checkpoint inhibition—Update 2019. *Eur. J. Cancer* **2020**, *130*, 126–138. https://doi.org/10.1016/j.ejca.2020.02.021.
9. Long, G.V.; Hodi, F.S.; Lipson, E.J.; Schadendorf, D.; Ascierto, P.A.; Matamala, L.; Salman, P.; Gutiérrez, E.C.; Rutkowski, P.; Gogas, H.; et al. Relatlimab and nivolumab versus nivolumab in previously untreated metastatic or unresectable melanoma: Overall survival and response rates from RELATIVITY-047 (CA224-047). *J. Clin. Oncol.* **2022**, *40*, 360385.
10. Long, G.V.; Luke, J.J.; Khattak, M.; de la Cruz Merino, L.; Del Vecchio, M.; Rutkowski, P.; Spagnolo, F.; Mackiewicz, J.; Chiarion-Sileni, V.; Kirkwood, J.M.; et al. Distant metastasis-free survival with pembrolizumab versus placebo as adjuvant therapy in stage IIB or IIC melanoma: The phase 3 KEYNOTE-716 study. *J. Clin. Oncol.* **2022**, *40*, LBA9500.
11. Long, G.V.D.V.; Weber, J.M.; Hoeller, C.; Grob, J.J.; Mohr, P.; Grabbe, S.; Dutriaux, C.; Chiarion-Sileni, V.; Mackiewicz, J. Adjuvant therapy with nivolumab versus placebo in patients with resected stage IIB/C melanoma (CheckMate 76K). In Proceedings of the SMR Meeting 2022, Edinburgh, UK, 17–20 October 2022.
12. Grossmann, K.F.; Othus, M.; Patel, S.P.; Tarhini, A.A.; Sondak, V.K.; Knopp, M.V.; Petrella, T.M.; Truong, T.G.; Khushalani, N.I.; Cohen, J.V.; et al. Adjuvant Pembrolizumab versus IFNalpha2b or Ipilimumab in Resected High-Risk Melanoma. *Cancer Discov.* **2022**, *12*, 644–653. https://doi.org/10.1158/2159-8290.CD-21-1141.
13. Larkin, J.; Del Vecchio, M.; Mandala, M.; Gogas, H.; Arance, A.M.; Dalle, S.; Cowey, C.L.; Schenker, M.; Grob, J.J.; Chiarion-Sileni, V.; et al. Adjuvant Nivolumab Versus Ipilimumab in Resected Stage III/IV Melanoma: 5-Year Efficacy and Biomarker Results from CheckMate 238. *Clin. Cancer Res.* **2023**, OF1. https://doi.org/10.1158/1078-0432.Ccr-22-3145.
14. Livingstone, E.; Zimmer, L.; Hassel, J.C.; Fluck, M.; Eigentler, T.K.; Loquai, C.; Haferkamp, S.; Gutzmer, R.; Meier, F.; Mohr, P.; et al. Adjuvant nivolumab plus ipilimumab or nivolumab alone versus placebo in patients with resected stage IV melanoma with no evidence of disease (IMMUNED): Final results of a randomised, double-blind, phase 2 trial. *Lancet* **2022**, *400*, 1117–1129. https://doi.org/10.1016/S0140-6736(22)01654-3.
15. Eggermont, A.M.M.; Kicinski, M.; Blank, C.U.; Mandala, M.; Long, G.V.; Atkinson, V.; Dalle, S.; Haydon, A.; Meshcheryakov, A.; Khattak, A.; et al. Five-Year Analysis of Adjuvant Pembrolizumab or Placebo in Stage III Melanoma. *NEJM Evid.* **2022**, *1*, EVIDoa2200214. https://doi.org/10.1056/EVIDoa2200214.
16. Guyatt, G.H.; Oxman, A.D.; Vist, G.E.; Kunz, R.; Falck-Ytter, Y.; Alonso-Coello, P.; Schunemann, H.J.; GRADE Working Group. GRADE: An emerging consensus on rating quality of evidence and strength of recommendations. *BMJ* **2008**, *336*, 924–926. https://doi.org/10.1136/bmj.39489.470347.AD.
17. Gajewski, T. Mechanisms of required resistance. In Proceedings of the ESMO Annual Meeting, Lugano, Switzerland, 9–13 September 2022.
18. Seymour, L.; Bogaerts, J.; Perrone, A.; Ford, R.; Schwartz, L.H.; Mandrekar, S.; Lin, N.U.; Litiere, S.; Dancey, J.; Chen, A.; et al. iRECIST: Guidelines for response criteria for use in trials testing immunotherapeutics. *Lancet Oncol.* **2017**, *18*, e143–e152. https://doi.org/10.1016/S1470-2045(17)30074-8.
19. Galldiks, N.; Kocher, M.; Ceccon, G.; Werner, J.M.; Brunn, A.; Deckert, M.; Pope, W.B.; Soffietti, R.; Le Rhun, E.; Weller, M.; et al. Imaging challenges of immunotherapy and targeted therapy in patients with brain metastases: Response, progression, and pseudoprogression. *Neuro Oncol.* **2020**, *22*, 17–30. https://doi.org/10.1093/neuonc/noz147.
20. Brandsma, D.; Stalpers, L.; Taal, W.; Sminia, P.; van den Bent, M.J. Clinical features, mechanisms, and management of pseudoprogression in malignant gliomas. *Lancet Oncol.* **2008**, *9*, 453–461. https://doi.org/10.1016/S1470-2045(08)70125-6.
21. Park, H.J.; Kim, K.W.; Pyo, J.; Suh, C.H.; Yoon, S.; Hatabu, H.; Nishino, M. Incidence of Pseudoprogression during Immune Checkpoint Inhibitor Therapy for Solid Tumors: A Systematic Review and Meta-Analysis. *Radiology* **2020**, *297*, 87–96. https://doi.org/10.1148/radiol.2020200443.
22. Borcoman, E.; Kanjanapan, Y.; Champiat, S.; Kato, S.; Servois, V.; Kurzrock, R.; Goel, S.; Bedard, P.; Le Tourneau, C. Novel patterns of response under immunotherapy. *Ann. Oncol.* **2019**, *30*, 385–396. https://doi.org/10.1093/annonc/mdz003.
23. Kluger, H.M.; Tawbi, H.A.; Ascierto, M.L.; Bowden, M.; Callahan, M.K.; Cha, E.; Chen, H.X.; Drake, C.G.; Feltquate, D.M.; Ferris, R.L.; et al. Defining tumor resistance to PD-1 pathway blockade: Recommendations from the first meeting of the SITC Immunotherapy Resistance Taskforce. *J. Immunother. Cancer* **2020**, *8*, e000398. https://doi.org/10.1136/jitc-2019-000398.
24. Beer, L.; Hochmair, M.; Haug, A.R.; Schwabel, B.; Kifjak, D.; Wadsak, W.; Fuereder, T.; Fabikan, H.; Fazekas, A.; Schwab, S.; et al. Comparison of RECIST, iRECIST, and PERCIST for the Evaluation of Response to PD-1/PD-L1 Blockade Therapy in Patients with Non-Small Cell Lung Cancer. *Clin. Nucl. Med.* **2019**, *44*, 535–543. https://doi.org/10.1097/RLU.0000000000002603.
25. Tetzlaff, M.T.; Messina, J.L.; Stein, J.E.; Xu, X.; Amaria, R.N.; Blank, C.U.; van de Wiel, B.A.; Ferguson, P.M.; Rawson, R.V.; Ross, M.I.; et al. Pathological assessment of resection specimens after neoadjuvant therapy for metastatic melanoma. *Ann. Oncol.* **2018**, *29*, 1861–1868. https://doi.org/10.1093/annonc/mdy226.
26. Brahmer, J.R.; Drake, C.G.; Wollner, I.; Powderly, J.D.; Picus, J.; Sharfman, W.H.; Stankevich, E.; Pons, A.; Salay, T.M.; McMiller, T.L.; et al. Phase I study of single-agent anti-programmed death-1 (MDX-1106) in refractory solid tumors: Safety, clinical activity, pharmacodynamics, and immunologic correlates. *J. Clin. Oncol.* **2010**, *28*, 3167–3175. https://doi.org/10.1200/JCO.2009.26.7609.
27. Jacquelot, N.; Yamazaki, T.; Roberti, M.P.; Duong, C.P.M.; Andrews, M.C.; Verlingue, L.; Ferrere, G.; Becharef, S.; Vetizou, M.; Daillere, R.; et al. Sustained Type I interferon signaling as a mechanism of resistance to PD-1 blockade. *Cell. Res.* **2019**, *29*, 846–861. https://doi.org/10.1038/s41422-019-0224-x.
28. Zaretsky, J.M.; Garcia-Diaz, A.; Shin, D.S.; Escuin-Ordinas, H.; Hugo, W.; Hu-Lieskovan, S.; Torrejon, D.Y.; Abril-Rodriguez, G.; Sandoval, S.; Barthly, L.; et al. Mutations Associated with Acquired Resistance to PD-1 Blockade in Melanoma. *N. Engl. J. Med.* **2016**, *375*, 819–829. https://doi.org/10.1056/NEJMoa1604958.
29. Zou, J.; Du, K.; Li, S.; Lu, L.; Mei, J.; Lin, W.; Deng, M.; Wei, W.; Guo, R. Glutamine Metabolism Regulators Associated with Cancer Development and the Tumor Microenvironment: A Pan-Cancer Multi-Omics Analysis. *Genes* **2021**, *12*, 1305. https://doi.org/10.3390/genes12091305.
30. Varghese, S.; Pramanik, S.; Williams, L.J.; Hodges, H.R.; Hudgens, C.W.; Fischer, G.M.; Luo, C.K.; Knighton, B.; Tan, L.; Lorenzi, P.L.; et al. The Glutaminase Inhibitor CB-839 (Telaglenastat) Enhances the Antimelanoma Activity of T-Cell-Mediated Immunotherapies. *Mol. Cancer Ther.* **2021**, *20*, 500–511. https://doi.org/10.1158/1535-7163.MCT-20-0430.
31. Trujillo, J.A.; Luke, J.J.; Zha, Y.; Segal, J.P.; Ritterhouse, L.L.; Spranger, S.; Matijevich, K.; Gajewski, T.F. Secondary resistance to immunotherapy associated with beta-catenin pathway activation or PTEN loss in metastatic melanoma. *J. Immunother. Cancer* **2019**, *7*, 295. https://doi.org/10.1186/s40425-019-0780-0.
32. Lee, J.H.; Shklovskaya, E.; Lim, S.Y.; Carlino, M.S.; Menzies, A.M.; Stewart, A.; Pedersen, B.; Irvine, M.; Alavi, S.; Yang, J.Y.H.; et al. Transcriptional downregulation of MHC class I and melanoma de- differentiation in resistance to PD-1 inhibition. *Nat. Commun.* **2020**, *11*, 1897. https://doi.org/10.1038/s41467-020-15726-7.
33. Syn, N.L.; Teng, M.W.L.; Mok, T.S.K.; Soo, R.A. De-novo and acquired resistance to immune checkpoint targeting. *Lancet Oncol.* **2017**, *18*, e731–e741. https://doi.org/10.1016/S1470-2045(17)30607-1.
34. Aldea, M.; Andre, F.; Marabelle, A.; Dogan, S.; Barlesi, F.; Soria, J.C. Overcoming Resistance to Tumor-Targeted and Immune-Targeted Therapies. *Cancer Discov.* **2021**, *11*, 874–899. https://doi.org/10.1158/2159-8290.CD-20-1638.
35. Wulfken, L.M.; Becker, J.C.; Hayajneh, R.; Wagner, A.D.; Schaper-Gerhardt, K.; Flatt, N.; Grimmelmann, I.; Gutzmer, R. Case Report: Sustained Remission Due to PD-1-Inhibition in a Metastatic Melanoma Patient with Depleted B Cells. *Front. Immunol.* **2021**, *12*, 733961. https://doi.org/10.3389/fimmu.2021.733961.
36. Ayers, M.; Lunceford, J.; Nebozhyn, M.; Murphy, E.; Loboda, A.; Kaufman, D.R.; Albright, A.; Cheng, J.D.; Kang, S.P.; Shankaran, V.; et al. IFN-gamma-related mRNA profile predicts clinical response to PD-1 blockade. *J. Clin. Investig.* **2017**, *127*, 2930–2940. https://doi.org/10.1172/JCI91190.
37. Blank, C.U.; Haanen, J.B.; Ribas, A.; Schumacher, T.N. Cancer Immunology. The “cancer immunogram”. *Science* **2016**, *352*, 658–660. https://doi.org/10.1126/science.aaf2834.
38. Wolchok, J.; Chiarion-Sileni, V.; Gonzalez, R.G.; Grob, J.J.; Rutkowski, P.; Lao, C.; Cowey, C.; Schadendorf, D.; Wagstaff, J.; Dummer, R.; et al. CheckMate 067: 6.5-year outcomes in patients (pts) with advanced melanoma. *J. Clin. Oncol.* **2021**, *39*, 9506.
39. Hamid, O.; Robert, C.; Daud, A.; Hodi, F.S.; Hwu, W.J.; Kefford, R.; Wolchok, J.D.; Hersey, P.; Joseph, R.; Weber, J.S.; et al. Five-year survival outcomes for patients with advanced melanoma treated with pembrolizumab in KEYNOTE-001. *Ann. Oncol.* **2019**, *30*, 582–588. https://doi.org/10.1093/annonc/mdz011.
40. Robert, C.; Long, G.V.; Brady, B.; Dutriaux, C.; Di Giacomo, A.M.; Mortier, L.; Rutkowski, P.; Hassel, J.C.; McNeil, C.M.; Kalinka, E.A.; et al. Five-Year Outcomes with Nivolumab in Patients with Wild-Type BRAF Advanced Melanoma. *J. Clin. Oncol.* **2020**, *38*, 3937–3946. https://doi.org/10.1200/JCO.20.00995.
41. Tawbi, H.A.; Forsyth, P.A.; Hodi, F.S.; Algazi, A.P.; Hamid, O.; Lao, C.D.; Moschos, S.J.; Atkins, M.B.; Lewis, K.; Postow, M.A.; et al. Long-term outcomes of patients with active melanoma brain metastases treated with combination nivolumab plus ipilimumab (CheckMate 204): Final results of an open-label, multicentre, phase 2 study. *Lancet Oncol.* **2021**, *22*, 1692–1704. https://doi.org/10.1016/S1470-2045(21)00545-3.
42. Long, G.V.; Atkinson, V.; Lo, S.; Guminski, A.D.; Sandhu, S.K.; Brown, M.P.; Gonzalez, M.; Scolyer, R.A.; Emmett, L.; McArthur, G.A.; et al. Five-year overall survival from the anti-PD1 brain collaboration (ABC Study): Randomized phase 2 study of nivolumab (nivo) or nivo+ipilimumab (ipi) in patients (pts) with melanoma brain metastases (mets). *J. Clin. Oncol.* **2021**, *39*, 9508.
43. Weide, B.M.A.; Hassel, J.C.; Berking, C.; Postow, M.A.; Bisschop, K.; Simeone, E.; Mangana, J.; Schilling, B.; Di Giacomo, A.M.; Brenner, N. Baseline Biomarkers for Outcome of Melanoma Patients Treated with Pembrolizumab. *Clin. Cancer Res.* **2016**, *22*, 5487–5496. https://doi.org/10.1158/1078-0432.CCR-16-0127.
44. Madonna, G.; Masucci, G.V.; Capone, M.; Mallardo, D.; Grimaldi, A.M.; Simeone, E.; Vanella, V.; Festino, L.; Palla, M.; Scarpato, L.; et al. Clinical Categorization Algorithm (CLICAL) and Machine Learning Approach (SRF-CLICAL) to Predict Clinical Benefit to Immunotherapy in Metastatic Melanoma Patients: Real-World Evidence from the Istituto Nazionale Tumori IRCCS Fondazione Pascale, Napoli, Italy. *Cancers* **2021**, *13*, 4164. https://doi.org/10.3390/cancers13164164.
45. Fattore, L.; Ruggiero, C.F.; Liguoro, D.; Castaldo, V.; Catizone, A.; Ciliberto, G.; Mancini, R. The Promise of Liquid Biopsy to Predict Response to Immunotherapy in Metastatic Melanoma. *Front. Oncol.* **2021**, *11*, 645069. https://doi.org/10.3389/fonc.2021.645069.
46. Weber, J.G.G.; Kudchadkar, R.; Yu, B.; Cheng, P.; Martinez, A.J.; Kroeger, J.; Richards, A.; McCormick, L.; Moberg, V.; Cronin, H. Phase I/II Study of Metastatic Melanoma Patients Treated with Nivolumab Who Had Progressed after Ipilimumab. *Cancer Immunol. Res.* **2016**, *4*, 345–353. https://doi.org/10.1158/2326-6066.CIR-15-0193.
47. Ugurel, S.; Schadendorf, D.; Horny, K.; Sucker, A.; Schramm, S.; Utikal, J.; Pfohler, C.; Herbst, R.; Schilling, B.; Blank, C.; et al. Elevated baseline serum PD-1 or PD-L1 predicts poor outcome of PD-1 inhibition therapy in metastatic melanoma. *Ann. Oncol.* **2020**, *31*, 144–152. https://doi.org/10.1016/j.annonc.2019.09.005.
48. Machiraju, D.; Wiecken, M.; Lang, N.; Hulsmeyer, I.; Roth, J.; Schank, T.E.; Eurich, R.; Halama, N.; Enk, A.; Hassel, J.C. Soluble immune checkpoints and T-cell subsets in blood as biomarkers for resistance to immunotherapy in melanoma patients. *Oncoimmunology* **2021**, *10*, 1926762. https://doi.org/10.1080/2162402X.2021.1926762.
49. Hassel, J.C.; Zucht, H.-D.; Mangana, J.; Dummer, R.; Pföhler, C.; Wistuba-Hamprecht, K.; Weide, B.; Hakim-Meibodi, L.E.; Meier, F.E.; Schulz, C.; et al. Autoantibodies as predictors for survival and immune-related adverse events in checkpoint inhibition therapy of metastasized melanoma. *J. Clin. Oncol.* **2020**, *38*, 10011.
50. Tawbi, H.A.; Schadendorf, D.; Lipson, E.J.; Ascierto, P.A.; Matamala, L.; Castillo Gutierrez, E.; Rutkowski, P.; Gogas, H.J.; Lao, C.D.; De Menezes, J.J.; et al. Relatlimab and Nivolumab versus Nivolumab in Untreated Advanced Melanoma. *N. Engl. J. Med.* **2022**, *386*, 24–34. https://doi.org/10.1056/NEJMoa2109970.
51. Chesney, J.A.; Ribas, A.; Long, G.V.; Kirkwood, J.M.; Dummer, R.; Puzanov, I.; Hoeller, C.; Gajewski, T.F.; Gutzmer, R.; Rutkowski, P.; et al. Randomized, Double-Blind, Placebo-Controlled, Global Phase III Trial of Talimogene Laherparepvec Combined with Pembrolizumab for Advanced Melanoma. *J. Clin. Oncol.* **2022**, *41*, 528–540. https://doi.org/10.1200/JCO.22.00343.
52. Diab, A.; Gogas, H.J.; Sandhu, S.K.; Long, G.V.; Ascierto, P.A.; Larkin, J.; Sznol, M.; Franke, F.A.; Ciuleanu, T.E.; Couselo, E.M.; et al. 785O—PIVOT IO 001: First disclosure of efficacy and safety of bempegaldesleukin (BEMPEG) plus nivolumab (NIVO) vs. NIVO monotherapy in advanced melanoma (MEL). *Ann. Oncol.* **2022**, *33*, S901.
53. Dummer, R.; Long, G.V.; Robert, C.; Tawbi, H.A.; Flaherty, K.T.; Ascierto, P.A.; Nathan, P.D.; Rutkowski, P.; Leonov, O.; Dutriaux, C.; et al. Randomized Phase III Trial Evaluating Spartalizumab Plus Dabrafenib and Trametinib for BRAF V600-Mutant Unresectable or Metastatic Melanoma. *J. Clin. Oncol.* **2022**, *40*, 1428–1438. https://doi.org/10.1200/JCO.21.01601.
54. Gutzmer, R.; Stroyakovskiy, D.; Gogas, H.; Robert, C.; Lewis, K.; Protsenko, S.; Pereira, R.P.; Eigentler, T.; Rutkowski, P.; Demidov, L.; et al. Atezolizumab, vemurafenib, and cobimetinib as first-line treatment for unresectable advanced BRAF(V600) mutation-positive melanoma (IMspire150): Primary analysis of the randomised, double-blind, placebo-controlled, phase 3 trial. *Lancet* **2020**, *395*, 1835–1844. https://doi.org/10.1016/S0140-6736(20)30934-X.
55. Ascierto, P.A.; Stroyakovskiy, D.; Gogas, H.; Robert, C.; Lewis, K.; Protsenko, S.; Pereira, R.P.; Eigentler, T.; Rutkowski, P.; Demidov, L.; et al. Overall survival with first-line atezolizumab in combination with vemurafenib and cobimetinib in BRAF(V600) mutation-positive advanced melanoma (IMspire150): Second interim analysis of a multicentre, randomised, phase 3 study. *Lancet Oncol.* **2022**, *24*, 33–44. https://doi.org/10.1016/S1470-2045(22)00687-8.
56. Sarnaik, A.; Khushalani, N.I.; Chesney, J.A.; Lewis, K.D.; Medina, T.M.; Kluger, H.M.; Thomas, S.S.; Domingo Musibay, E.; Pavlick, A.C.; Whitman, E.D.; et al. Long-term follow up of lifileucel (LN-144) cryopreserved autologous tumor infiltrating lymphocyte therapy in patients with advanced melanoma progressed on multiple prior therapies. *J. Clin. Oncol.* **2020**, *38*, 10006.
57. Arance, A.; de la Cruz-Merino, L.; Petrella, T.M.; Jamal, R.; Ny, L.; Carneiro, A.; Berrocal, A.; Marquez-Rodas, I.; Spreafico, A.; Atkinson, V.; et al. Phase II LEAP-004 Study of Lenvatinib Plus Pembrolizumab for Melanoma with Confirmed Progression on a Programmed Cell Death Protein-1 or Programmed Death Ligand 1 Inhibitor Given as Monotherapy or in Combination. *J. Clin. Oncol.* **2023**, *41*, 75–85. https://doi.org/10.1200/JCO.22.00221.
58. Long, G.V.; Atkinson, V.; Lo, S.; Sandhu, S.; Guminski, A.D.; Brown, M.P.; Wilmott, J.S.; Edwards, J.; Gonzalez, M.; Scolyer, R.A.; et al. Combination nivolumab and ipilimumab or nivolumab alone in melanoma brain metastases: A multicentre randomised phase 2 study. *Lancet Oncol.* **2018**, *19*, 672–681. https://doi.org/10.1016/S1470-2045(18)30139-6.
59. Olson, D.; Luke, J.J.; Poklepovic, A.S.; Bajaj, M.; Higgs, E.; Carll, T.C.; Labadie, B.; Krausz, T.; Zha, Y.; Karrison, T.; et al. Significant antitumor activity for low-dose ipilimumab (IPI) with pembrolizumab (PEMBRO) immediately following progression on PD1 Ab in melanoma (MEL) in a phase II trial. *J. Clin. Oncol.* **2020**, *38*, 10004.
60. Ascierto, P.A.; Bono, P.; Bhatia, S.; Melero, I.; Nyakas, M.S.; Svane, I.M.; Larkin, J.; Gomez-Roca, C.; Schadendorf, D.; Dummer, R.; et al. Efficacy of BMS-986016, a monoclonal antibody that targets lymphocyte activation gene-3 (LAG-3), in combination with nivolumab in pts with melanoma who progressed during prior anti–PD-1/PD-L1 therapy (mel prior IO) in all-comer and biomarker-enriched populations. *Ann. Oncol.* **2017**, *28*, v611–v612.
61. Amin, A.; Milhem, M.M.; Long, G.V.; Hoimes, C.J.; Medina, T.M.; Conry, R.M.; Lao, C.D.; Daniels, G.A.; Reddy, S.A.; Andtbacka, R.H.I.; et al. Phase 1b/2, open label, multicenter, study of the combination of SD-101 and pembrolizumab in patients with advanced/metastatic melanoma resistant to anti-PD-1/PD-L1 therapy. *J. Clin. Oncol.* **2019**, *37*.
62. Amaria, R.N.; Haymaker, C.L.; Forget, M.-A.; Bassett, R.; Cormier, J.N.; Davies, M.A.; Diab, A.; Gershenwald, J.E.; Glitza, I.C.; Lee, J.E.; et al. Lymphodepletion (LD), tumor-infiltrating lymphocytes (TIL) and high (HD-IL2) versus low-dose (LD-IL2) IL-2 followed by pembrolizumab (pembro) in patients (pts) with metastatic melanoma (MM). *J. Clin. Oncol.* **2019**, *37*, 9543.
63. Zager, J.S.; Sarnaik, A.S.; Pilon-Thomas, S.; Beatty, M.; Han, D.; Lu, G.; Agarwala, S.S.; Ross, M.; Shirai, K.; Essner, R.; et al. 1123P A phase Ib study of rose bengal disodium and anti-PD-1 in metastatic cutaneous melanoma: Initial results in patients refractory to checkpoint blockade. *Ann. Oncol.* **2020**, *31*, S755–S756.
64. Hassel, J.C.; Berking, C.; Schlaak, M.; Eigentler, T.; Gutzmer, R.; Ascierto, P.A.; Schilling, B.; Hamm, S.; Hermann, F.; Reimann, P.G.; et al. Results from the phase Ib of the SENSITIZE trial combining domatinostat with pembrolizumab in advanced melanoma patients refractory to prior checkpoint inhibitor therapy. *J. Clin. Oncol.* **2021**, *39*, 9545.
65. McCarter, M.; Tobin, R.P.; Cogswell, D.T.; Vorwald, V.M.; Davis, D.; Van Gulick, R.J.; Couts, K.L.; Jordan, K.R.; Nuanes, V.; Gao, D.; et al. Pembrolizumab and all-trans retinoic acid combination treatment of advanced melanoma. *J. Clin. Oncol.* **2021**, *39*, 9536.
66. Loquai, C.; Hassel, J.C.; Oehm, P.; Derhovanessian, E.; Jabulowsky, R.A.; Gold, M.; Schwarck-Kokarakis, D.; Attig, S.; Cuk, K.; Vogler, I.; et al. A shared tumor-antigen RNA-lipoplex vaccine with/without anti-PD1 in patients with checkpoint-inhibition experienced melanoma. *J. Clin. Oncol.* **2020**, *38*, 3136.
67. Sahin, U.; Oehm, P.; Derhovanessian, E.; Jabulowsky, R.A.; Vormehr, M.; Gold, M.; Maurus, D.; Schwarck-Kokarakis, D.; Kuhn, A.N.; Omokoko, T.; et al. An RNA vaccine drives immunity in checkpoint-inhibitor-treated melanoma. *Nature* **2020**, *585*, 107–112. https://doi.org/10.1038/s41586-020-2537-9.
68. Haymaker, C.; Johnson, D.H.; Murthy, R.; Bentebibel, S.E.; Uemura, M.I.; Hudgens, C.W.; Safa, H.; James, M.; Andtbacka, R.H.I.; Johnson, D.B.; et al. Tilsotolimod with Ipilimumab Drives Tumor Responses in Anti-PD-1 Refractory Melanoma. *Cancer Discov.* **2021**, *11*, 1996–2013. https://doi.org/10.1158/2159-8290.CD-20-1546.
69. Idera Pharmaceuticals*. Idera Pharmaceuticals Announces Results from Illuminate-301 Trial of Tilsotolimod + Ipilimumab in Anti-Pd-1 Refractory Advanced Melanoma*; Idera Pharmaceuticals, Inc.: Exton, PA, USA, 2021.
70. Pires da Silva, I.; Ahmed, T.; Reijers, I.L.M.; Weppler, A.M.; Betof Warner, A.; Patrinely, J.R.; Serra-Bellver, P.; Allayous, C.; Mangana, J.; Nguyen, K.; et al. Ipilimumab alone or ipilimumab plus anti-PD-1 therapy in patients with metastatic melanoma resistant to anti-PD-(L)1 monotherapy: A multicentre, retrospective, cohort study. *Lancet Oncol.* **2021**, *22*, 836–847. https://doi.org/10.1016/S1470-2045(21)00097-8.
71. Rohaan, M.W.; Borch, T.H.; van den Berg, J.H.; Met, O.; Kessels, R.; Geukes Foppen, M.H.; Stoltenborg Granhoj, J.; Nuijen, B.; Nijenhuis, C.; Jedema, I.; et al. Tumor-Infiltrating Lymphocyte Therapy or Ipilimumab in Advanced Melanoma. *N. Engl. J. Med.* **2022**, *387*, 2113–2125. https://doi.org/10.1056/NEJMoa2210233.
72. Atkins, M.B.; Lee, S.J.; Chmielowski, B.; Tarhini, A.A.; Cohen, G.I.; Truong, T.G.; Moon, H.H.; Davar, D.; O’Rourke, M.; Stephenson, J.J.; et al. Combination Dabrafenib and Trametinib Versus Combination Nivolumab and Ipilimumab for Patients with Advanced BRAF-Mutant Melanoma: The DREAMseq Trial-ECOG-ACRIN EA6134. *J. Clin. Oncol.* **2023**, *41*, 186–197. https://doi.org/10.1200/jco.22.01763.
73. Kabiljo, J.; Harpain, F.; Carotta, S.; Bergmann, M. Radiotherapy as a Backbone for Novel Concepts in Cancer Immunotherapy. *Cancers* **2019**, *12*, 79. https://doi.org/10.3390/cancers12010079.
74. Guckenberger, M.; Lievens, Y.; Bouma, A.B.; Collette, L.; Dekker, A.; de Souza, N.M.; Dingemans, A.C.; Fournier, B.; Hurkmans, C.; Lecouvet, F.E.; et al. Characterisation and classification of oligometastatic disease: A European Society for Radiotherapy and Oncology and European Organisation for Research and Treatment of Cancer consensus recommendation. *Lancet Oncol.* **2020**, *21*, e18–e28. https://doi.org/10.1016/S1470-2045(19)30718-1.
75. Versluis, J.M.; Hendriks, A.M.; Weppler, A.M.; Brown, L.J.; de Joode, K.; Suijkerbuijk, K.P.M.; Zimmer, L.; Kapiteijn, E.W.; Allayous, C.; Johnson, D.B.; et al. The role of local therapy in the treatment of solitary melanoma progression on immune checkpoint inhibition: A multicentre retrospective analysis. *Eur. J. Cancer* **2021**, *151*, 72–83. https://doi.org/10.1016/j.ejca.2021.04.003.
76. Zaremba, A.; Eggermont, A.M.M.; Robert, C.; Dummer, R.; Ugurel, S.; Livingstone, E.; Ascierto, P.A.; Long, G.V.; Schadendorf, D.; Zimmer, L. The concepts of rechallenge and retreatment with immune checkpoint blockade in melanoma patients. *Eur. J. Cancer* **2021**, *155*, 268–280. https://doi.org/10.1016/j.ejca.2021.07.002.
77. Robert, C.C.; Carlino, M.S.; McNeil, C.; Rebas, A.; Grob, J.J.; Schachter, J.; Nyakas, M.; Kee, D.; Petrella, T.; Blaustein, A.; et al. 7-year Follow-up of KEYNOTE-006: Pembrolizumab (pembro) Versus Ipilimumab (ipi) in Advanced Melanoma. *Pigment. Cell. Melanoma Res.* **2022**, *35*, 97–184.
78. Hassel, J.C. 5-year results for pembrolizumab treatment of advanced melanoma. *Lancet Oncol.* **2019**, *20*, 1187–1189. https://doi.org/10.1016/S1470-2045(19)30483-8.
79. Betof Warner, A.; Palmer, J.S.; Shoushtari, A.N.; Goldman, D.A.; Panageas, K.S.; Hayes, S.A.; Bajwa, R.; Momtaz, P.; Callahan, M.K.; Wolchok, J.D.; et al. Long-Term Outcomes and Responses to Retreatment in Patients with Melanoma Treated With PD-1 Blockade. *J. Clin. Oncol.* **2020**, *38*, 1655–1663. https://doi.org/10.1200/JCO.19.01464.
80. Reschke, R.; Simon, J.C.; Ziemer, M. Rechallenge of targeted therapy in metastatic melanoma. *J. Dtsch. Dermatol. Ges.* **2019**, *17*, 483–486. https://doi.org/10.1111/ddg.13766.
81. Owen, C.N.; Shoushtari, A.N.; Chauhan, D.; Palmieri, D.J.; Lee, B.; Rohaan, M.W.; Mangana, J.; Atkinson, V.; Zaman, F.; Young, A.; et al. Management of early melanoma recurrence despite adjuvant anti-PD-1 antibody therapy. *Ann. Oncol.* **2020**, *31*, 1075–1082. https://doi.org/10.1016/j.annonc.2020.04.471.
82. Gutzmer, R.; Vordermark, D.; Hassel, J.C.; Krex, D.; Wendl, C.; Schadendorf, D.; Sickmann, T.; Rieken, S.; Pukrop, T.; Holler, C.; et al. Melanoma brain metastases—Interdisciplinary management recommendations 2020. *Cancer Treat. Rev.* **2020**, *89*, 102083. https://doi.org/10.1016/j.ctrv.2020.102083.
83. Engelhardt, B.; Ransohoff, R.M. Capture, crawl, cross: The T cell code to breach the blood-brain barriers. *Trends Immunol.* **2012**, *33*, 579–589. https://doi.org/10.1016/j.it.2012.07.004.
84. Davies, M.A.; Saiag, P.; Robert, C.; Grob, J.J.; Flaherty, K.T.; Arance, A.; Chiarion-Sileni, V.; Thomas, L.; Lesimple, T.; Mortier, L.; et al. Dabrafenib plus trametinib in patients with BRAF(V600)-mutant melanoma brain metastases (COMBI-MB): A multicentre, multicohort, open-label, phase 2 trial. *Lancet Oncol.* **2017**, *18*, 863–873. https://doi.org/10.1016/s1470-2045(17)30429-1.
85. Welti, M.; Dimitriou, F.; Gutzmer, R.; Dummer, R. Triple Combination of Immune Checkpoint Inhibitors and BRAF/MEK Inhibitors in BRAFV600 Melanoma: Current Status and Future Perspectives. *Cancers* **2022**, *14*, 5489. https://doi.org/10.3390/cancers14225489.
86. Matsunaga, S.; Shuto, T.; Yamamoto, M.; Yomo, S.; Kondoh, T.; Kobayashi, T.; Sato, M.; Okamoto, H.; Serizawa, T.; Nagano, O.; et al. Gamma Knife Radiosurgery for Metastatic Brain Tumors from Malignant Melanomas: A Japanese Multi-Institutional Cooperative and Retrospective Cohort Study (JLGK1501). *Stereotact. Funct. Neurosurg.* **2018**, *96*, 162–171. https://doi.org/10.1159/000489948.
87. Kessel, K.A.; Deichl, A.; Gempt, J.; Meyer, B.; Posch, C.; Diehl, C.; Zimmer, C.; Combs, S.E. Outcomes after stereotactic radiosurgery of brain metastases in patients with malignant melanoma and validation of the melanoma molGPA. *Clin. Transl. Oncol.* **2021**, *23*, 2020–2029. https://doi.org/10.1007/s12094-021-02607-8.
88. Zhong, J.; Press, R.H.; Olson, J.J.; Oyesiku, N.M.; Shu, H.G.; Eaton, B.R. The use of Hypofractionated Radiosurgery for the Treatment of Intracranial Lesions Unsuitable for Single-Fraction Radiosurgery. *Neurosurgery* **2018**, *83*, 850–857. https://doi.org/10.1093/neuros/nyy145.
89. Gaudy-Marqueste, C.; Dussouil, A.S.; Carron, R.; Troin, L.; Malissen, N.; Loundou, A.; Monestier, S.; Mallet, S.; Richard, M.A.; Regis, J.M.; et al. Survival of melanoma patients treated with targeted therapy and immunotherapy after systematic upfront control of brain metastases by radiosurgery. *Eur. J. Cancer* **2017**, *84*, 44–54. https://doi.org/10.1016/j.ejca.2017.07.017.
90. Lehrer, E.J.; McGee, H.M.; Sheehan, J.P.; Trifiletti, D.M. Integration of immuno-oncology with stereotactic radiosurgery in the management of brain metastases. *J. Neurooncol.* **2021**, *151*, 75–84. https://doi.org/10.1007/s11060-020-03427-6.
91. Martins, F.; Schiappacasse, L.; Levivier, M.; Tuleasca, C.; Cuendet, M.A.; Aedo-Lopez, V.; Gautron Moura, B.; Homicsko, K.; Bettini, A.; Berthod, G.; et al. The combination of stereotactic radiosurgery with immune checkpoint inhibition or targeted therapy in melanoma patients with brain metastases: A retrospective study. *J. Neurooncol.* **2020**, *146*, 181–193. https://doi.org/10.1007/s11060-019-03363-0.
92. Liermann, J.; Winkler, J.K.; Syed, M.; Neuberger, U.; Reuss, D.; Harrabi, S.; Naumann, P.; Ristau, J.; Weykamp, F.; El Shafie, R.A.; et al. Stereotactic Radiosurgery with Concurrent Immunotherapy in Melanoma Brain Metastases Is Feasible and Effective. *Front. Oncol.* **2020**, *10*, 592796. https://doi.org/10.3389/fonc.2020.592796.
93. Rauschenberg, R.; Bruns, J.; Brutting, J.; Daubner, D.; Lohaus, F.; Zimmer, L.; Forschner, A.; Zips, D.; Hassel, J.C.; Berking, C.; et al. Impact of radiation, systemic therapy and treatment sequencing on survival of patients with melanoma brain metastases. *Eur. J. Cancer* **2019**, *110*, 11–20. https://doi.org/10.1016/j.ejca.2018.12.023.
94. Tetu, P.; Allayous, C.; Oriano, B.; Dalle, S.; Mortier, L.; Leccia, M.T.; Guillot, B.; Dalac, S.; Dutriaux, C.; Lacour, J.P.; et al. Impact of radiotherapy administered simultaneously with systemic treatment in patients with melanoma brain metastases within MelBase, a French multicentric prospective cohort. *Eur. J. Cancer* **2019**, *112*, 38–46. https://doi.org/10.1016/j.ejca.2019.02.009.
95. Amaral, T.; Kiecker, F.; Schaefer, S.; Stege, H.; Kaehler, K.; Terheyden, P.; Gesierich, A.; Gutzmer, R.; Haferkamp, S.; Uttikal, J.; et al. Combined immunotherapy with nivolumab and ipilimumab with and without local therapy in patients with melanoma brain metastasis: A DeCOG* study in 380 patients*. J. Immunother. Cancer* **2020**, *8*, e000333. https://doi.org/10.1136/jitc-2019-000333.
96. Borius, P.Y.; Regis, J.; Carpentier, A.; Kalamarides, M.; Valery, C.A.; Latorzeff, I. Safety of radiosurgery concurrent with systemic therapy (chemotherapy, targeted therapy, and/or immunotherapy) in brain metastases: A systematic review. *Cancer Metastasis Rev.* **2021**, *40*, 341–354. https://doi.org/10.1007/s10555-020-09949-9.
97. Jiang, J.M.; Kabarriti, R.; Brodin, N.P.; Ohri, N.; Guha, C.; Kalnicki, S.; Garg, M. Stereotactic radiosurgery with immunotherapy is associated with improved overall survival in patients with metastatic melanoma or non-small cell lung cancer: A National Cancer Database analysis. *Clin. Transl. Oncol.* **2022**, *24*, 104–111. https://doi.org/10.1007/s12094-021-02675-w.
98. Chen, G.; Chakravarti, N.; Aardalen, K.; Lazar, A.J.; Tetzlaff, M.T.; Wubbenhorst, B.; Kim, S.B.; Kopetz, S.; Ledoux, A.A.; Gopal, Y.N.; et al. Molecular profiling of patient-matched brain and extracranial melanoma metastases implicates the PI3K pathway as a therapeutic target. *Clin. Cancer Res.* **2014**, *20*, 5537–5546. https://doi.org/10.1158/1078-0432.CCR-13-3003.
99. Niessner, H.; Forschner, A.; Klumpp, B.; Honegger, J.B.; Witte, M.; Bornemann, A.; Dummer, R.; Adam, A.; Bauer, J.; Tabatabai, G.; et al. Targeting hyperactivation of the AKT survival pathway to overcome therapy resistance of melanoma brain metastases. *Cancer Med.* **2013**, *2*, 76–85. https://doi.org/10.1002/cam4.50.
100. Niessner, H.; Schmitz, J.; Tabatabai, G.; Schmid, A.M.; Calaminus, C.; Sinnberg, T.; Weide, B.; Eigentler, T.K.; Garbe, C.; Schittek, B.; et al. PI3K Pathway Inhibition Achieves Potent Antitumor Activity in Melanoma Brain Metastases In Vitro and In Vivo. *Clin. Cancer Res.* **2016**, *22*, 5818–5828. https://doi.org/10.1158/1078-0432.CCR-16-0064.
101. Tehranian, C.; Fankhauser, L.; Harter, P.N.; Ratcliffe, C.D.H.; Zeiner, P.S.; Messmer, J.M.; Hoffmann, D.C.; Frey, K.; Westphal, D.; Ronellenfitsch, M.W.; et al. The PI3K/Akt/mTOR pathway as a preventive target in melanoma brain metastasis. *Neuro Oncol.* **2022**, *24*, 213–225. https://doi.org/10.1093/neuonc/noab159.
102. Peng, W.; Chen, J.Q.; Liu, C.; Malu, S.; Creasy, C.; Tetzlaff, M.T.; Xu, C.; McKenzie, J.A.; Zhang, C.; Liang, X.; et al. Loss of PTEN Promotes Resistance to T Cell-Mediated Immunotherapy. *Cancer Discov.* **2016**, *6*, 202–216. https://doi.org/10.1158/2159-8290.CD-15-0283.
103. Fischer, G.M.; Jalali, A.; Kircher, D.A.; Lee, W.C.; McQuade, J.L.; Haydu, L.E.; Joon, A.Y.; Reuben, A.; de Macedo, M.P.; Carapeto, F.C.L.; et al. Molecular Profiling Reveals Unique Immune and Metabolic Features of Melanoma Brain Metastases. *Cancer Discov.* **2019**, *9*, 628–645. https://doi.org/10.1158/2159-8290.CD-18-1489.
104. Fischer, G.M.; Guerrieri, R.A.; Hu, Q.; Joon, A.Y.; Kumar, S.; Haydu, L.E.; McQuade, J.L.; Vashisht Gopal, Y.N.; Knighton, B.; Deng, W.; et al. Clinical, molecular, metabolic, and immune features associated with oxidative phosphorylation in melanoma brain metastases. *Neurooncol. Adv.* **2021**, *3*, vdaa177. https://doi.org/10.1093/noajnl/vdaa177.
105. Amaral, T.; Niessner, H.; Sinnberg, T.; Thomas, I.; Meiwes, A.; Garbe, C.; Garzarolli, M.; Rauschenberg, R.; Eigentler, T.; Meier, F. An open-label, single-arm, phase II trial of buparlisib in patients with melanoma brain metastases not eligible for surgery or radiosurgery-the BUMPER study. *Neurooncol. Adv.* **2020**, *2*, vdaa140. https://doi.org/10.1093/noajnl/vdaa140.
106. Ascierto, P.A.; Del Vecchio, M.; Mandala, M.; Gogas, H.; Arance, A.M.; Dalle, S.; Cowey, C.L.; Schenker, M.; Grob, J.J.; Chiarion-Sileni, V.; et al. Adjuvant nivolumab versus ipilimumab in resected stage IIIB-C and stage IV melanoma (CheckMate 238): 4-year results from a multicentre, double-blind, randomised, controlled, phase 3 trial. *Lancet Oncol.* **2020**, *21*, 1465–1477. https://doi.org/10.1016/S1470-2045(20)30494-0.
107. Eggermont, A.M.M.; Blank, C.U.; Mandala, M.; Long, G.V.; Atkinson, V.G.; Dalle, S.; Haydon, A.M.; Meshcheryakov, A.; Khattak, A.; Carlino, M.S.; et al. Longer Follow-Up Confirms Recurrence-Free Survival Benefit of Adjuvant Pembrolizumab in High-Risk Stage III Melanoma: Updated Results from the EORTC 1325-MG/KEYNOTE-054 Trial. *J. Clin. Oncol.* **2020**, *38*, 3925–3936. https://doi.org/10.1200/JCO.20.02110.
108. Zimmer, L.; Livingstone, E.; Hassel, J.C.; Fluck, M.; Eigentler, T.; Loquai, C.; Haferkamp, S.; Gutzmer, R.; Meier, F.; Mohr, P.; et al. Adjuvant nivolumab plus ipilimumab or nivolumab monotherapy versus placebo in patients with resected stage IV melanoma with no evidence of disease (IMMUNED): A randomised, double-blind, placebo-controlled, phase 2 trial. *Lancet* **2020**, *395*, 1558–1568. https://doi.org/10.1016/S0140-6736(20)30417-7.
109. Long, G.V.; Schadendorf, D.; Vecchio, M.D.; Larkin, J.; Atkinson, V.; Schenker, M.; Pigozzo, J.; Gogas, H.J.; Dalle, S.; Meyer, N.; et al. Adjuvant therapy with nivolumab (NIVO) combined with ipilimumab (IPI) vs NIVO alone in patients (pts) with resected stage IIIB-D/IV melanoma (CheckMate 915). *Cancer Res.* **2021**, *81*, CT004.
110. Weber, J.S.; Schadendorf, D.; Del Vecchio, M.; Larkin, J.; Atkinson, V.; Schenker, M.; Pigozzo, J.; Gogas, H.; Dalle, S.; Meyer, N.; et al. Adjuvant Therapy of Nivolumab Combined with Ipilimumab Versus Nivolumab Alone in Patients with Resected Stage IIIB-D or Stage IV Melanoma (CheckMate 915). *J. Clin. Oncol.* **2022**, *41*, 517–527. https://doi.org/10.1200/JCO.22.00533.
111. Eggermont, A.M.M.; Blank, C.U.; Mandala, M.; Long, G.V.; Atkinson, V.G.; Dalle, S.; Haydon, A.M.; Meshcheryakov, A.; Khattak, A.; Carlino, M.S.; et al. Adjuvant pembrolizumab versus placebo in resected stage III melanoma (EORTC 1325-MG/KEYNOTE-054): Distant metastasis-free survival results from a double-blind, randomised, controlled, phase 3 trial. *Lancet Oncol.* **2021**, *22*, 643–654. https://doi.org/10.1016/S1470-2045(21)00065-6.
112. Luke, J.J.; Rutkowski, P.; Queirolo, P.; Del Vecchio, M.; Mackiewicz, J.; Sileni, V.C.; de la Cruz Merino, L.; Khattak, M.A.; Schadendorf, D.; Long, G.V.; et al. LBA3_PR—Pembrolizumab versus placebo after complete resection of high-risk stage II melanoma: Efficacy and safety results from the KEYNOTE-716 double-blind phase III trial. *Ann. Oncol.* **2021**, *32*, S1314–S1315.
113. Long, G.V.; Desai, K.; Tang, T.; Weber, J.S.; Dolfi, S.; Ritchings, C.; Huang, S.P.; Bolisetty, M.; Sausen, M.; Del Vecchio, M.; et al. 788O Association of pre-treatment ctDNA with disease recurrence and clinical and translational factors in patients with stage IIIB-D/IV melanoma treated with adjuvant immunotherapy (CheckMate 915). *Ann. Oncol.* **2022**, *33*, S904.
114. Weber, J.S.; Del Vecchio, M.; Mandala, M.; Gogas, H.; Arance, A.M.; Dalle, S.; Cowey, C.L.; Schenker, M.; Grob, J.J.; Chiarion-Sileni, V.; et al. 1310O—Adjuvant nivolumab (NIVO) versus ipilimumab (IPI) in resected stage III/IV melanoma: 3-year efficacy and biomarker results from the phase III CheckMate 238 trial. *Ann. Oncol.* **2019**, *30*, v533–v534.
115. Gebhardt, C.; Ascierto, P.; Atkinson, V.; Corrie, P.; Dummer, R.; Schadendorf, D. The concepts of rechallenge and retreatment in melanoma: A proposal for consensus definitions. *Eur. J. Cancer* **2020**, *138*, 68–76. https://doi.org/10.1016/j.ejca.2020.07.016.
116. Eggermont, A.M.; Meshcheryakov, A.; Atkinson, V.; Blank, C.U.; Mandala, M.; Long, G.V.; Barrow, C.; Di Giacomo, A.M.; Fisher, R.; Sandhu, S.; et al. Crossover and rechallenge with pembrolizumab in recurrent patients from the EORTC 1325-MG/Keynote-054 phase III trial, pembrolizumab versus placebo after complete resection of high-risk stage III melanoma. *Eur. J. Cancer* **2021**, *158*, 156–168. https://doi.org/10.1016/j.ejca.2021.09.023.
117. Menzies, A.M.; Amaria, R.N.; Rozeman, E.A.; Huang, A.C.; Tetzlaff, M.T.; van de Wiel, B.A.; Lo, S.; Tarhini, A.A.; Burton, E.M.; Pennington, T.E.; et al. Pathological response and survival with neoadjuvant therapy in melanoma: A pooled analysis from the International Neoadjuvant Melanoma Consortium (INMC). *Nat. Med.* **2021**, *27*, 301–309. https://doi.org/10.1038/s41591-020-01188-3.
118. Amaria, R.N.; Postow, M.A.; Tetzlaff, M.T.; Ross, M.I.; Glitza, I.C.; McQuade, J.L.; Wong, M.K.K.; Gershenwald, J.E.; Goepfert, R.; Keung, E.Z.-Y.; et al. Neoadjuvant and adjuvant nivolumab (nivo) with anti-LAG3 antibody relatlimab (rela) for patients (pts) with resectable clinical stage III melanoma. *J. Clin. Oncol.* **2021**, *39*, 9502.
119. Amaria, R.N.; Reddy, S.M.; Tawbi, H.A.; Davies, M.A.; Ross, M.I.; Glitza, I.C.; Cormier, J.N.; Lewis, C.; Hwu, W.J.; Hanna, E.; et al. Neoadjuvant immune checkpoint blockade in high-risk resectable melanoma. *Nat. Med.* **2018**, *24*, 1649–1654. https://doi.org/10.1038/s41591-018-0197-1.
120. Kendra, K.L.; Moon, J.; Eroglu, Z.; Hu-Lieskovan, S.; Carson, W.E.; Wada, D.A.; Plaza, J.A.; In, G.K.; Ikeguchi, A.; Hyngstrom, J.R.; et al. Neoadjuvant PD-1 blockade in patients with resectable desmoplastic melanoma (SWOG 1512). *J. Clin. Oncol.* **2022**, *40*, 9502.
121. Eroglu, Z.; Zaretsky, J.M.; Hu-Lieskovan, S.; Kim, D.W.; Algazi, A.; Johnson, D.B.; Liniker, E.; Ben, K.; Munhoz, R.; Rapisuwon, S.; et al. High response rate to PD-1 blockade in desmoplastic melanomas. *Nature* **2018**, *553*, 347–350. https://doi.org/10.1038/nature25187.
122. Blank, C.U.; Rozeman, E.A.; Fanchi, L.F.; Sikorska, K.; van de Wiel, B.; Kvistborg, P.; Krijgsman, O.; van den Braber, M.; Philips, D.; Broeks, A.; et al. Neoadjuvant versus adjuvant ipilimumab plus nivolumab in macroscopic stage III melanoma. *Nat. Med.* **2018**, *24*, 1655–1661. https://doi.org/10.1038/s41591-018-0198-0.
123. Patel, S.O.; Othus, M.; Prieto, V.; Lowe, M.; Buchbinder, E.; Chen, Y.; Hyngstrom, J.; Lao, C.D.; Truong, T.; Chandra, S.; et al. LBA6—Neoadjvuant versus adjuvant pembrolizumab for resected stage III-IV melanoma (SWOG S1801). *Ann. Oncol.* **2022**, *33*, S1408.
124. Buder-Bakhaya, K.; Hassel, J.C. Biomarkers for Clinical Benefit of Immune Checkpoint Inhibitor Treatment-A Review from the Melanoma Perspective and Beyond. *Front. Immunol.* **2018**, *9*, 1474. https://doi.org/10.3389/fimmu.2018.01474.
125. Subrahmanyam, P.B.; Dong, Z.; Gusenleitner, D.; Giobbie-Hurder, A.; Severgnini, M.; Zhou, J.; Manos, M.; Eastman, L.M.; Maecker, H.T.; Hodi, F.S. Distinct predictive biomarker candidates for response to anti-CTLA-4 and anti-PD-1 immunotherapy in melanoma patients. *J. Immunother. Cancer* **2018**, *6*, 18. https://doi.org/10.1186/s40425-018-0328-8.
126. Ouwerkerk, W.; van den Berg, M.; van der Niet, S.; Limpens, J.; Luiten, R.M. Biomarkers, measured during therapy, for response of melanoma patients to immune checkpoint inhibitors: A systematic review. *Melanoma Res.* **2019**, *29*, 453–464. https://doi.org/10.1097/CMR.0000000000000589.
127. Amaria, R.N.; Menzies, A.M.; Burton, E.M.; Scolyer, R.A.; Tetzlaff, M.T.; Antdbacka, R.; Ariyan, C.; Bassett, R.; Carter, B.; Daud, A.; et al. Neoadjuvant systemic therapy in melanoma: Recommendations of the International Neoadjuvant Melanoma Consortium. *Lancet Oncol.* **2019**, *20*, e378–e389. https://doi.org/10.1016/S1470-2045(19)30332-8.
128. Mushti, S.L.; Mulkey, F.; Tang, S.; Singh, H.; Lemery, S.J.; Goldberg, K.B.; Sridhara, R.; Keegan, P.; Kluetz, P.G.; Pazdur, R.; et al. Immune Response Evaluation and Treatment with Immune Checkpoint Inhibitors Beyond Clinical Progression: Response Assessments for Cancer Immunotherapy. *Curr. Oncol. Rep.* **2020**, *22*, 116. https://doi.org/10.1007/s11912-020-00974-z.
