# Supplementary material for: Medical Needs and Therapeutic Options for Melanoma Patients Resistant to Anti-PD-1-Directed Immune Checkpoint Inhibition
Source: Cancers (Basel). 2023 Jun 30;15(13):3448. doi: 10.3390/cancers15133448 (PMC10341224; doi:10.3390/cancers15133448)
Supplement: Supplementary file 1 [file cancers-15-03448-s001.zip › cancers-2446945-supplementary.pdf]

Supplementary Materials: Medical Needs and Therapeutic Options for Melanoma Patients Resistant to Anti-PD-1 Directed Immune Checkpoint Inhibition

Jessica C. Hassel, Lisa Zimmer, Thomas Sickmann, Thomas K. Eigentler, Friedegund Meier, Peter Mohr, Tobias Pukrop, Alexander Roesch, Dirk Vordermark, Christina Wendl and Ralf Gutzmer

Table S1. Frequencies of response and resistance in selected neoadjuvant immunotherapy trials in melanoma (ICI monotherapy, or combined ICI).

Trial	Treatment Regimens	Trial Phase	Patients	N	pCR, %	Near pCR, %	pPR, %	pNR, %	MPR %	ORR % RECIST	CR, % RECIST	PR, % RECIST	SD, % RECIST	PD, % RECIST
NeoTrio [Long, ASCO 2022] (NCT02858921)	**(i)** Pem 200 mg q3w x 2<br>**(ii)** Dab (D) + Tram (T) q1w, Pem 200 mg day 8+22<br>**(iii)** D (150mg bid) + T (2mg qd) + Pem 200mg q3w x 2	II	Stage III resect. lymph nodes, no in transit, BRAF V600 mutated	**60** (20/arm)	6 (30%)<br>3 (15%)<br>10 (50%)	2 (10%)<br>3 (15%)<br>1 (5%)	3 (15%)<br>4 (20%)<br>5 (25%)	7 (35%)<br>10 (50%)<br>3 (15%)	40<br>30<br>55	30<br>45<br>70	2 (10%)<br>0<br>7 (35%)	4 (20%)<br>9 (45%)<br>7 (35%)	5 (25%)<br>1 (5%)<br>1 (5%)	9 (45%)<br>10 (50%)<br>5 (25%)
DONIMI [Blank, ESMO 2021] (NCT04133948)	**(i)** IFNg high: Nivo<br>**(ii)** IFNg high: Nivo + Domatinostat<br>**(iii)** IFNg low: Nivo + Domatinostat<br>**(iv)** IFNg low: Ipi + Nivo + Domatinostat	Ib	Stage III resectable	**40** (10/arm)	7 (70%)<br>5 (50%)<br>1 (10%)<br>3 (30%)	1 (10%)<br>1 (10%)<br>0<br>1 (10%)	1 (10%)<br>2 (20%)<br>2 (20%)<br>0	1 (10%)<br>2 (20%)<br>7 (70%<br>6 (60%)	80<br>60<br>10<br>40	50<br>70<br>0<br>40	1 (10%)<br>3 (30%)<br>0<br>1 (10%)	4 (40%)<br>4 (40%)<br>0<br>3 (30%)	5 (50%)<br>3 (30%)<br>9 (90%)<br>1 (10%)	0<br>0<br>0<br>5 (50%)
OpACIN [Blank, Nature Med 2018] (NCT02437279)	(i) Ipi 3mg/kg + Nivo 1mg/kg q3w x2	Ib	Stage III resectable	**8**	2 (25%)	3 (37%)	1 (12%)	2 (25%)	62	50	0	4 (50%)	3 (37%)	1 (13%)
OpACIN-NEO [Rozeman, ESMO 2018] (NCT02977052)	Ipi 3mg/kg + Nivo 1mg/kg<br>Ipi 1mg/kg + Nivo 3mg/kg<br>Ipi 3mg/kg q3w x2, than Nivo 3mg/kg<br><u>All three regimen</u>: q3w x2	II	Stage III resectable	**(i) 30**<br>**(ii) 30**<br>**(iii) 26**	14 (47%)<br>17 (57%)<br>6 (23%)	-<br>-<br>-	7 (23%)<br>2 (7%)<br>15 (17%)	6 (20%)<br>7 (23%)<br>8 (31%)	70<br>64<br>40	60<br>60<br>42	2 (7%)<br>3 (10%)<br>1 (4%)	16 (53%)<br>15 (50%)<br>10 (38%)	10 (33%)<br>9 (30%)<br>9 (35%)	2 (7%)<br>2 (7%)<br>6 (23%)
PRADO [Rozeman, ASCO 2020] (NCT02977052)	Ipi 1mg/kg + Nivo 3mg/kg q3w x2	II	Stage III resectable	**99**	49 (50%)	11 (11%)	10 (10%)	21 (21%)	61	45	14 (14%)	31 (31%)	38 (38%)	13 (13%)
2015-0041 [Amaria ASCO 2021] (NCT02519322)	Rela 160mg+ Nivo 480mg q4w x2	II	Stage III resectable	**30**	17 (59%)	2 (7%)	2 (7%)	8 (27%)	66	57	0	17 (57%)	10 (33%)	3 (10%)
2015-0041 [Amaria, Nature Med 2018] (NCT02519322)	**(i)** Nivo 3mg/kg q2w x4<br>(ii) Nivo 1mg/kg + Ipi 3mg/kg q3w x4x	II	Stage III or IV resectable	**12**<br>**11**	3 (25%)<br>5 (45%)	-	-	-	-	25<br>73	0<br>1 (9%)	3 (25%)<br>7 (64%)	5 (42%)<br>2 (18%)	4 (33%)<br>1 (9%)
UPCC 01615 [Huang, Nature Med 2019] (NCT02434354)	Pem 200mg flat, single-dose	IB	Stage III or IV resectable	**27**	5 (18%)	3 (11%)	5 (18%)	14 (52%)	29	50	0	3/6 (50%)	1/6 (17%)	2/6 (33%)
SWOG1512 [Kendra, ASCO 2022] (NCT02775851)	Pem 200mg q3w x3	II	Resectable desmoplastic melanoma	**29**	16 (55%)	-	-	-	-	46	4 (15%)	8 (31%)	12 (46%)	1 (4%)

Abbreviations: Dab, dabrafenib; ICI, immune checkpoint inhibitor; Ipi, ipilimumab; MPR, major pathological response; Nivo, nivolumab; Pem, pembrolizumab; Tram, trametinib; x1 (,x2, …), once (twice, …).
